# The transcriptional response of *Pasteurella multocida *to three classes of antibiotics

**DOI:** 10.1186/1471-2164-10-S2-S4

**Published:** 2009-07-14

**Authors:** Bindu Nanduri, Leslie A Shack, Shane C Burgess, Mark L Lawrence

**Affiliations:** 1 College of Veterinary Medicine, Mississippi State University, Mississippi State, MS 39762, USA; 2 Institute for Digital Biology, Mississippi State University, Mississippi State, MS 39762, USA; 3 Life Sciences and Biotechnology Institute, Mississippi State University, Mississippi State, MS 39762, USA; 4 Mississippi Agriculture and Forestry Experiment Station, Mississippi State University, Mississippi State, MS 39762, USA

## Abstract

**Background:**

*Pasteurella multocida *is a gram-negative bacterial pathogen that has a broad host range. One of the diseases it causes is fowl cholera in poultry. The availability of the genome sequence of avian *P. multocida *isolate Pm70 enables the application of functional genomics for observing global gene expression in response to a given stimulus. We studied the effects of three classes of antibiotics on the *P. multocida *transcriptome using custom oligonucleotide microarrays from NimbleGen Systems. Hybridizations were conducted with RNA isolated from three independent cultures of Pm70 grown in the presence or absence of sub-minimum inhibitory concentration (sub-MIC) of antibiotics. Differentially expressed (DE) genes were identified by ANOVA and Dunnett's test. Biological modeling of the differentially expressed genes (DE) was conducted based on Clusters of Orthologous (COG) groups and network analysis in Pathway Studio.

**Results:**

The three antibiotics used in this study, amoxicillin, chlortetracycline, and enrofloxacin, collectively influenced transcription of 25% of the *P. multocida *Pm70 genome. Some DE genes identified were common to more than one antibiotic. The overall transcription signatures of the three antibiotics differed at the COG level of the analysis. Network analysis identified differences in the SOS response of *P. multocida *in response to the antibiotics.

**Conclusion:**

This is the first report of the transcriptional response of an avian strain of *P. multocida *to sub-lethal concentrations of three different classes of antibiotics. We identified common adaptive responses of *P. multocida *to antibiotic stress. The observed changes in gene expression of known and putative *P. multocida *virulence factors establish the molecular basis for the therapeutic efficacy of sub-MICs. Our network analysis demonstrates the feasibility and limitations of applying systems modeling to high throughput datasets in 'non-model' bacteria.

## Background

*Pasteurella multocida *is a gram–negative bacterial pathogen that has a unique history of serving as a model species for new discoveries. In his seminal experiments in the late eighteenth century, Louis Pasteur demonstrated both attenuation as well as protective immune response utilizing *P. multocida *in birds [1,2]. *P. multocida *is also an excellent model species for studying the effects of antibiotics on gram-negative bacteria because its short lipopolysaccharide O side chains make it more permeable, allowing investigations on the effects of antibiotics on bacterial metabolism [3].

Of the different *P. multocida *subspecies, multocida has a broad host range and causes diseases in poultry, cattle, pigs, and rabbits [4]. Zoonosis with *P. multocida *is caused by cat and dog bites and scratches and respiratory infection [5]. Fowl cholera (avian pasteurellosis) caused by *P. multocida *can be either chronic or acute and is a septicemic disease that causes significant economic loss to the poultry industry. Due to the importance of this pathogen to the avian community, the genome of an isolate recovered from chicken fowl cholera (strain Pm70) was sequenced in 2001 [6]. The genome sequence has enabled subsequent functional genomics research with this species [7-9]. It has also enabled investigations on the proteomic and transcriptomic response of *P. multocida *Pm70 to sub-MICs of antibiotics [10-12].

Much attention has been focused on characterization of specific antibiotic resistance mechanisms; however, recent studies are shedding light on non-target effects of antibiotics. In the current study, we used *P. multocida *as a model species to investigate the effects of three antibiotics with disparate mechanisms of action (amoxicillin, chlortetracycline, and enrofloxacin) on bacterial metabolism. Because biological systems utilize highly complex, interrelated metabolic and signaling networks, a thorough understanding of bacterial physiology requires studying the global response to a given stimulus in the context of interacting gene networks. Computational systems biology facilitates this level of analysis. Systems level analysis can identify key regulatory elements of a molecular interaction network; dynamic changes in these elements govern a response [13].

Application of systems approaches to *P. multocida *is still in the initial stages, but we have started using these methods to characterize the *P. multocida *response to antibiotics at the proteome level [11]. Here we describe the use of systems analysis at the transcriptome level to investigate the *P. multocida *response to sub-MICs of antibiotics.

## Results and discussion

### Differentially expressed genes

**Figure 1:**
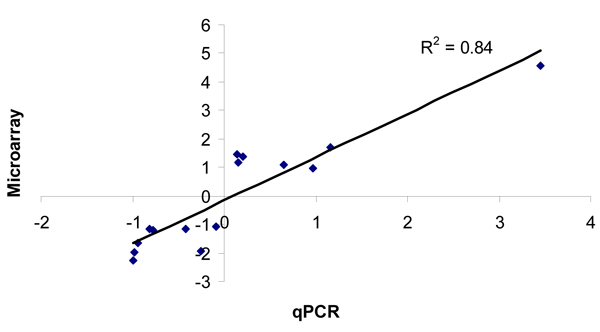
**Validation of microarray differential gene expression data by quantitative real time RT-PCR (qPCR)**. Ratio of treated vs. control were calculated for microarray data (ordinate) and qPCR data (abscissa). A total of six genes that were up or down regulated with amoxicillin, chlortetracycline or enrofloxacin were compared. The correlation coefficient r^2^ was 0.84.

Statistical analysis of microarray data revealed that 1/4 MIC of AMX, CTC, and ENR resulted in significant changes in expression of approximately 25% of the genome (525 genes, Additional file 1, supplemental table 1). The differences in gene expression that were determined to be statistically significant ranged from as small as a 5% decrease (relative to control) to as high as an 11.5-fold increase in expression (*recN*, Table 1). Earlier microarray studies with *P. multocida *arbitrarily chose a 1.5-fold significant change in gene expression as differentially regulated genes. Instead of choosing a pre-determined threshold for determining the biological relevance of changes in gene expression, we considered every significant change in expression to be a valid change based on our rigorous statistical testing. We validated changes as small as a 15% decrease or increase (for example, expression of *murA *in response to AMX and ENR, respectively) (Additional file 1, supplemental table 1) by qPCR. Figure 1 shows the correlation between the trends observed in microarray and qPCR for the subset of genes with qPCR validations.

**Table 1:** Differential expression of *P. multocida *genes involved in SOS response

GeneID	Product Name	Locus	AMX*	CTC*	ENR*
1244239	ImpA	impA	-0.52	-0.53	4.55
1244433	DNA-binding protein Fis	fis	ns	ns	1.41
1244528	LexA repressor	lexA	ns	1.60	9.87
1244611	Hypothetical protein PM1264	-	-0.78	-0.57	ns
1244637	Transcriptional repressor protein MetJ	metJ	-0.53	-0.74	-0.80
1244659	Hypothetical protein PM1312	-	-0.56	-0.68	-0.31
1244737	DNA-directed RNA polymerase subunit alpha	rpoA	ns	-0.77	1.27
1244795	Hypothetical protein PM1448	-	-0.81	-0.70	1.29
1244828	Hypothetical protein PM1481	-	-0.53	-0.51	-0.51
1244993	Hypothetical protein PM1646	-	-0.62	ns	-0.70
1245061	Hypothetical protein PM1714	-	-0.74	-0.91	ns
1245083	DNA-directed RNA polymerase subunit beta'	rpoC	ns	-0.76	-0.87
1245184	ATP-dependent helicase HepA	hepA	-0.77	-0.77	1.69
1245314	DNA-binding transcriptional activator GutM	gutM	-0.55	-0.69	-0.62
1243539	Hypothetical protein PM0192	recJ	-0.77	-0.79	1.24
1243679	Hypothetical protein PM0332	recN	ns	ns	11.54
1243758	DNA-dependent helicase II	uvrD	ns	ns	4.26
1243814	DNA polymerase IV	dinP	-0.76	-0.83	1.83
1244020	Hypothetical protein PM0673	-	-0.63	-0.81	-0.47
1244323	Holliday junction DNA helicase B	ruvB	ns	-0.78	2.37
1244324	Holliday junction DNA helicase motor protein	ruvA	ns	ns	3.39
1244459	DeaD	deaD	-0.78	-0.72	-0.67
1244499	DNA repair protein RadC	radC	-0.52	-0.60	-0.65
1244507	DNA polymerase III subunit beta	dnaN	ns	ns	1.41
1244526	Primosomal replication protein N	priB	ns	ns	1.67
1244563	DNA polymerase III subunit delta	holA	ns	-0.88	1.38
1244666	MutY	mutY	-0.68	-0.86	1.17
1244774	RecQ	recQ	-0.86	-0.84	1.11
1244823	DNA gyrase subunit B	gyrB	ns	1.36	1.45
1245063	NAD-dependent DNA ligase LigA	ligA	-0.84	1.08	1.56
1245164	Recombinase A	recA	ns	ns	9.96
1245177	DNA mismatch repair protein	mutS	-0.87	-0.78	1.50
1245268	ATP-dependent RNA helicase RhlB	rhlB	-0.73	-0.83	1.07
1245298	Excinuclease ABC subunit A	uvrA	-0.78	ns	2.57
1243785	Hypothetical protein PM0438	ftsH	-0.94	-0.80	1.13
1244083	Hypothetical protein PM0736	DnaK	ns	-0.74	1.50
1244087	Hypothetical protein PM0740	dnaJ	-0.74	-0.79	1.29
1245051	ClpB	clpB	-0.66	ns	2.14
1245095	ATP-dependent protease ATP-binding subunit	hslU	-0.70	ns	2.58
1245163	Recombination regulator RecX	recX	-0.54	-0.78	6.44

* Ratio of treatment/control normalized intensities. ns: no significant change in gene expression with sub-MIC of antibiotic.

**Figure 2:**
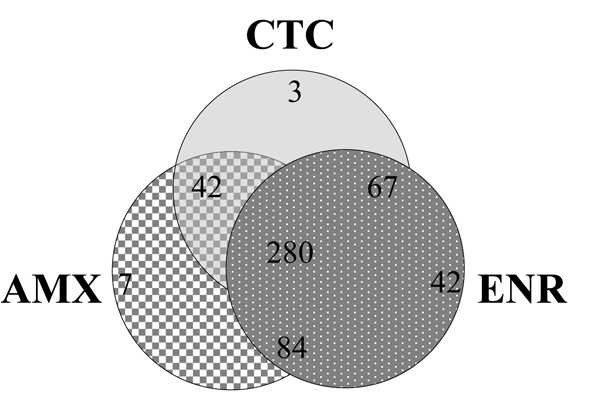
**Venn diagram of gene expression in response to sub-MIC antibiotics**. The data intersections between significant changes in gene expression of *P. multocida *in response to sub-MIC amoxicillin (AMX), chlortetracycline (CTC) and enrofloxacin (ENR).

Compared to the untreated control, expression of 413, 392, and 473 genes had significantly altered expression in response to AMX, CTC, and ENR, respectively, and the overlap between treatments is shown in the Venn diagram (Figure 2). The extensive overlap of regulated genes with the three different antibiotics is consistent with the recent transcriptome analysis of ampicillin and ofloxacin effects in *E. coli*, which showed that these two unrelated antibiotics had a significant overlap of regulated genes [14].

### Functional analysis of DE genes: COGs

**Figure 3:**
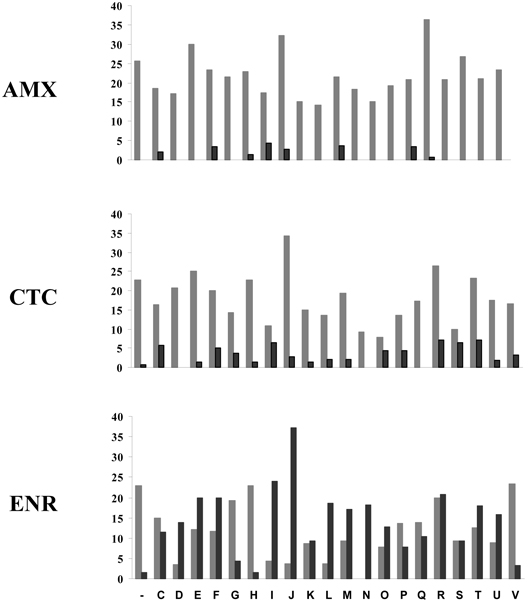
**Summary of significant changes in *P. multocida *gene expression grouped by COGs**. Numbers on the y-axis represent the percentage of genes in each COG category whose expression either significantly increased (black bars) or decreased (gray bars) in response to antibiotic treatment. COG category descriptions are: A, RNA processing and modification; K, Transcription; L, Replication, recombination and repair; D, Cell cycle control, mitosis and meiosis; V, Defense mechanisms; T, Signal transduction mechanisms; M, Cell wall/membrane biogenesis; N, Cell motility; W, Extracellular structures; U, Intracellular trafficking and secretion; O, Posttranslational modification, protein turnover, chaperones; C, Energy production and conversion; G, Carbohydrate transport and metabolism; E, Amino acid transport and metabolism; F, Nucleotide transport and metabolism; H, Coenzyme transport and metabolism; I, Lipid transport and metabolism P, Inorganic ion transport and metabolism; Q, Secondary metabolites biosynthesis, transport and catabolism R, General function prediction only; S, Function unknown; -, not in COGs

The three antibiotics used in this study have distinct modes of action; AMX is a cell wall biosynthesis inhibitor, CTC is a protein synthesis inhibitor, and ENR inhibits DNA gyrase and DNA topoisomerase IV activities [15]. At the COG category level, the three antibiotics had different effects on gene expression (Figure 3). The AMX and CTC treatments resulted in an overall decrease in gene expression in all COG categories. The overall suppression of gene expression could indicate that the antibiotics have a marked detrimental effect on the fitness of *P. multocida *at doses below MIC, or it is also possible that the overall transcriptional shutdown is a compensatory response by slowing metabolism. ENR had varying effects on gene expression in different COG categories. In particular, ENR caused a pronounced increase in expression of genes in categories I (lipid metabolism), J (translation, ribosomal structure and biogenesis), and L (DNA replication, recombination and repair). All three antibiotics had reduced expression of genes in COGs V (defense mechanisms), G (carbohydrate transport and metabolism), P (inorganic ion transport and metabolism) and Q (secondary metabolites biosynthesis, transport and catabolism).

### Effects of antibiotics on known target genes

We observed altered expression of some of the known targets (direct or inferred based on mechanism of action) with each antibiotic. CTC reduced the expression of genes encoding ribosomal proteins S4 and S7, which interact with tetracyclines [16]. One quarter-MIC of ENR resulted in a 1.5 fold increase in the expression of *gyrB*, which encodes a known ENR target, DNA gyrase B (Table 2). We had previously reported that the expression of RecA protein increased in response to sub-MIC of ENR [10], and in the current study we detected increased expression of *recA*. With AMX, except for the expression of *ponC*, there were no detectable differences in the expression of genes encoding penicillin binding proteins (pbp). There was a 50% decrease in *ponC *(pbp) gene expression. However, this was a not an AMX specific effect, as all three antibiotics caused a decrease in *ponC *gene expression.

**Table 2:** Changes in specific target gene expression in response to sub-MIC of antibiotics

GeneID	Product Name	Locus	AMX*	CTC*	ENR*	COG(s)
1244738	30S ribosomal protein S4	rpsD	ns	-0.82	1.29	COG0522J
1244702	30S ribosomal protein S7	rpS7	1.11	-0.93	1.69	COG0049J
1244823	DNA gyrase subunit B	gyrB	ns	1.36	1.45	COG0187L
1245164	Recombinase A	recA	ns	ns	9.96	COG0468L
1243991	PonC	ponC	-0.55	-0.56	-0.62	COG4953M

*Ratio of treatment/control normalized intensities. ns: no significant change in gene expression with sub-MIC of antibiotic.

While regulation of expression of known targets is an expected response to antibiotics, it is not a consistent finding; expression of the target is usually not affected. For example, microarray analysis showed that enrofloxacin, trimethoprim, brodimoprim, and cefquinome had no effects on expression of their targets in *P. multocida *[12]. However, even though expression of the known antibiotic target is often not affected, bacteria typically have a "signature" gene expression response to specific antibiotics. Indeed, bacterial transcription profiles are now sometimes used to obtain initial indications of the mechanism of action for new compounds [17,18].

### Common trends in response to AMX, CTC and ENR

Despite differences in their mechanism of action, the majority of genes with significantly altered expression (282) were common to all three antibiotics (Figure 2). Beyond the effects on specific target gene expression, antibiotics are known to cause secondary effects on genes involved in general physiology as part of the adaptive response to antibiotic stress. A hallmark of recently described antibiotic mediated bacterial cell death common to bactericidal antibiotics in *E. coli *is the generation of hydroxyl radicals with subsequent induction of SOS response [19,20]. These effects were noted for MIC as well as sub-MIC concentrations of the antibiotics. There is increasing appreciation for the fact that fine tuning of these responses is unique to each organism and needs to be evaluated as such to facilitate identification of targets that potentiate the bactericidal activity of antibiotics. The *P. multocida *genome is known to contain genes necessary for antibiotic mediated SOS response [6] (for example, *sulA*, which is necessary for beta-lactam mediated SOS response).

Antibiotic stress in *P. multocida *resulted in increased ATP synthesis (Additional file 2, supplemental table 2). While this effect has been described for gyrase inhibitors [21], in *P. multocida *this response was common to all three antibiotics. In particular, all three antibiotics influenced gene expression involved in *de novo *nucleotide biosynthesis. The overall effects on expression of *purM *(increase), *purK *(decrease), and *purF *(decrease) could reduce the intracellular concentrations of IMP, while increased expression of *pyrG *and *pyrF *with ENR could result in increased concentrations of CTP and UDP.

Both AMX and CTC impaired the expression of *hsp90*, thus affecting proper protein folding under stress conditions in *P. multocida*. All three classes of antibiotics adversely affected putrescine (*potE*), ribose (*rbscA *and *rbscC*), and molybdate (*modC*) transport systems. Alterations to *modC *expression could have wide ranging effects on bacterial physiology due to effects on the synthesis of molybdoenzymes (Additional file 2, supplemental table 2). Reduced levels of polyamines like putrescine could have profound negative effects on protein translation and impair the ability to cope with oxidative stress [22].

Expression of 121 genes annotated as coding for hypothetical proteins were altered in response to the antibiotics (Additional file 1, supplemental table 1). Lack of functional information for these genes hampers our understanding of their role in adaptation of *P. multocida *to antibiotic stress and demonstrates the need for continuous functional annotation of genes beyond the initial annotation that is described with the genome sequence.

One of the demonstrated outcomes of antibiotic stress in *E. coli *is alterations in iron homeostasis; namely, expression of genes involved in siderophore mediated iron transport are affected (for example, *fecC*) [19]. In *P. multocida*, expression of genes encoding iron transporters *fecC *and *fecE *were reduced when it was grown in the presence of 1/4 MIC of AMX or ENR (Additional file 2, supplemental table 2).

Changes in the ratio of NAD+/NADH are known to influence the levels of toxic superoxide formation, which ultimately leads to hydroxyl radical formation. NADH I levels are directly linked to the activities of TCA cycle enzymes that generate NADH from NAD+ [19]. ENR treatment caused decreased expression of *sucB*, and all three antibiotics caused decreased *lpdA *expression, which could reduce hydroxyl radical formation. Expression of the gene encoding anaerobic respiration electron transfer enzyme molybdoenzyme dimethylsulfoxide reductase was reduced in response to AMX and ENR. AMX treatment reduced the expression of acetyl-CoA carboxylase enzyme (*accB, accD*), which catalyzes the first step of type II fatty acid biosynthesis as well as the step catalyzed by acyl carrier protein S-malonyltransferase (*fabD*); by contrast, ENR increased the expression of these genes, which could result in increased fatty acid biosynthesis.

### DE genes: network analysis

COG analysis of DE genes was useful for identifying overall trends at a broad functional category level. Gene ontology (GO) analysis of *P. multocida*, which is available through Uniprot, was useful to some extent in adding specific functions to the genes within each of the broad functional categories represented by COGs. However, neither of these two approaches could actually show exactly how the genes within a functional category were interacting with each other to bring about a specific function. To enable network analysis, we used Pathway Studio to build interaction networks for all DE *P. multocida *genes with AMX, CTC and ENR. Careful analysis of these networks revealed expression cascades for some of the DE genes common to CTC and ENR.

**Figure 4:**
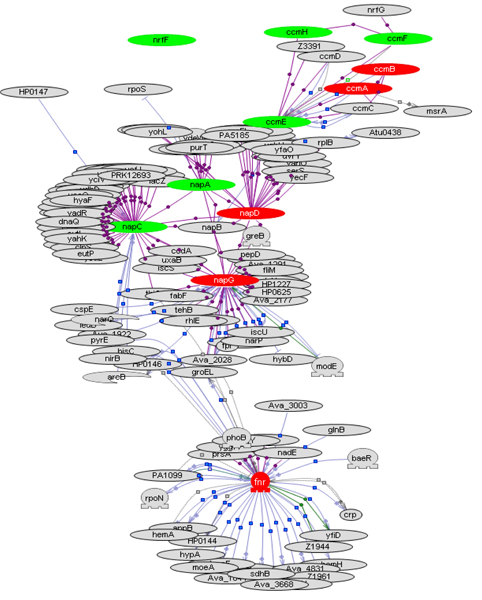
**Fumarate/nitrate response regulator network in *P. multocida *in response to enrofloxacin visualized in Pathway Studio**. *P. multocida *sub-MIC ENR response was marked by significant changes in *fnr *response regulated genes. Red nodes are genes with increased expression and green nodes are genes with decreased expression. The rest of the interacting nodes are shown in gray. These nodes had no significant changes in expression or were orthologs from additional gram-negative species in the molecular interaction database in Pathway Studio.

Expression of *fnr *increased in response to sub-MIC of CTC and ENR (Figure 4). Fnr regulates the transcription of many genes that are involved in either aerobic or anaerobic respiration, including Nap nitrate reductase, which facilitates nitrate reduction in the periplasmic space. Expression of *napD *and *napF *increased in response to ENR and decreased with CTC (Additional file 3, supplemental figure 1). NapD is a chaperone protein that is specific for NapA and is required for the stability of NapA protein. NapF stimulates the Nap reductase. NapF promoters regulate the transcription of *cmABCDEFGH *operon in *E. coli*, which constitutes the cytochrome biogenesis system. Expression of *ccmH *increased with CTC and decreased with ENR (Table 3). The identification of this cascade of gene regulation was possible by combining the network analysis with the functional information available at EcoCyc. The implications of up regulation of enzymes that facilitate anaerobic respiration in response to antibiotics remain unclear at present.

**Table 3:** Fnr regulated genes and their response to sub-MIC of chlortetracycline and enrofloxacin

GeneID	Product Name	Locus	CTC*	ENR*
1244015	Fumarate/nitrate reduction transcriptional regulator	fnr	1.49	1.43
1243352	Cytochrome c biogenesis protein CcmA	ccmA	-0.70	1.80
1243353	CcmB	ccmB	-0.45	1.27
1243356	cytochrome c-type biogenesis protein CcmE	ccmE	-0.77	-0.57
1243357	CcmF	ccmF	ns	-0.44
1243359	CcmH	ccmH_1	1.41	-0.43
1243360	CcmH	ccmH_2	1.56	-0.52
1244939	Hypothetical protein PM1592	napF	-0.50	2.11
1244940	NapD	napD	-0.52	1.35
1244941	Nitrate reductase	napA	-0.81	-0.76
1244945	NapC	napC	1.40	-0.68

* Ratio of treatment/control normalized intensities. ns: no significant change in gene expression with sub-MIC of antibiotic.

**Figure 5:**
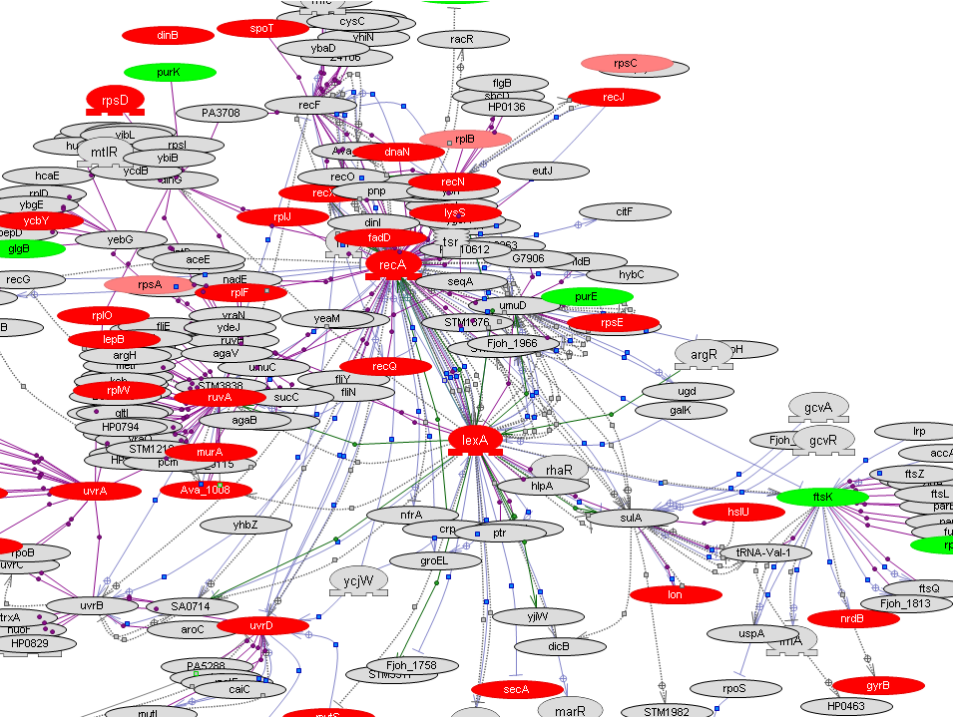
**SOS response gene network in *P. multocida *in response to enrofloxacin**. *P. multocida *sub-MIC ENR response was marked by significant changes in SOS response genes. Red nodes are genes with increased expression and green nodes are genes with decreased expression. Pink nodes represent no significant changes in gene expression while gray nodes are orthologs from gram-negative species in the molecular interaction database in Pathway Studio.

Network analysis also facilitated the identification of differences in the SOS response with different antibiotics in *P. multocida *(Figure 5 (ENR) and Additional file 3, supplemental figures 2 (AMX) and 3 (CTC)). In particular, the response to ENR showed the signature gene expression pattern typical of SOS response (Figure 5). Increased RecA protein expression results in the cleavage of LexA repressor, thus removing the LexA mediated repression of DNA repair genes like *uvrD*. Expression of both *recA *and *lexA *genes increased in response to ENR (Table 1). Expression of *recN *was increased, which also relieves LexA repression [23-25]. Genes involved in fine-tuning the actions of RecA, namely *recX *and *uvrD*, also had increased expression. Expression of primosome genes (*dnaJ *and *dnaK*) that encode enzymes required for re-starting the stalled replication fork and other replicative enzymes like DNA polymerase III increased with ENR.

It is reported that beta lactam mediated cell killing involves SOS response through SulA and DpiBA two component system [26]. Based on the genome annotation of Pm70 [6], all the components of this regulon are not present. There was no induction of the expression of *recA *or *lexA *with AMX (Additional file 3, supplemental figure 2). The expression profile of SOS response genes remained either unchanged or showed decreased expression, which indicates that sub-MIC AMX does not activate SOS response in *P*. *multocida*. Excluding the increased expression of *lexA *and *gyrB*, CTC (Additional file 3, supplemental figure 3) also did not activate SOS response.

### DE virulence genes

Productive infection by bacterial pathogens relies on the expression of virulence factors that have wide ranging functions like competence, adherence, capsule synthesis and export, evading host immune responses etc. Transcription profiling of the response of a bovine *P. multocida *isolate (L386) to MIC of eight different antibiotics identified mostly reduced virulence gene expression [12]. In the current study, transcription profiling of the *P. multocida *response to AMX, CTC and ENR identified significant changes in the expression of known and putative virulence genes. AMX and CTC reduced the expression of *ompA*, a known virulence factor of *P. multocida *involved in binding to host cells [27] (Table 4). AMX and CTC decreased expression of the gene encoding detoxifying enzyme superoxide dismutase, which is one of the virulence genes identified in the *P. multocida *L386 study [12]. Additional virulence factors (based on L386 study) which had reduced expression in Pm70 are: *clpB *protease with AMX, capsule transport protein *hexA *with all three antibiotics, PM1714 regulator with AMX and CTC, capsule biosynthesis genes *phyA and hyaE *with AMX and CTC, *phyB *with CTC, and tight adherence gene *tadB *with AMX. Expression of *dnaK*, which is required for virulence in a number of bacterial pathogens [26], was decreased by CTC.

**Table 4:** Effects of sub-MIC of antibiotics on *P. multocida *putative virulence factors

GeneID	Product Name	Locus	AMX*	CTC*	ENR*
1244083	Hypothetical protein PM0736	DnaK	ns	-0.74	1.50
1244119	PhyB	phyB	ns	-0.84	1.41
1244120	PhyA	phyA	-0.80	-0.71	1.71
1244121	HyaE	hyaE	-0.64	-0.91	1.27
1244128	HexA	hexA	-0.74	-0.69	-0.73
1244133	Hypothetical protein PM0786	OmpA	-0.83	-0.75	ns
1244195	Hypothetical protein PM0848	tadB	-0.51	ns	ns
1245051	ClpB	clpB	-0.66	ns	2.14
1245061	Hypothetical protein PM1714	-	-0.74	-0.91	ns

* Ratio of treatment/control normalized intensities. ns: no significant change in gene expression with sub-MIC of antibiotic.

## Conclusion

Our global transcriptome analysis of *P. multocida *response to three antibiotics with differing modes of action identified gene expression changes in a little over a quarter of the annotated open reading frames in the genome. The antibiotics had varying effects on various cellular and metabolic functions. Amoxicillin reduced the overall transcription rate as reflected by the majority of the identified significant changes in gene expression showing a downward trend in expression. An interesting aspect of the AMX response was the marked lack of expression of SOS response genes. Enrofloxacin, a DNA gyrase inhibitor, resulted in the significant overexpression of its target gene and induced a typical SOS response. Analysis of DE genes in the context of interacting protein networks facilitated the identification of coordinated regulation of gene expression across different COG functional categories. Sub-MIC of antibiotics influenced the expression of virulence factors in *P. multocida*. Our results form the framework for understanding the global effects of antibiotics on *P. multocida*, which will aid rational drug design for containing as well as treating infections caused by this pathogen across multiple species.

## Methods

### Bacterial culture and RNA isolation

*P. multocida *Pm70 was cultivated in brain heart infusion broth (BHI) at 37°C with rotary aeration. Minimum inhibitory concentrations (MICs) of AMX, CTC and ENR for Pm70 are 0.5 μg/ml, 4 μg/ml, and 0.031 μg/ml, respectively [10]. Growth kinetics of Pm70 in the presence of 1/4 MIC of the three antibiotics were previously described [10]. Stationary phase cultures of Pm70 were used to inoculate 50 ml BHI to an initial A_600 _of 0.05; antibiotic treated cultures contained 1/4 MIC of AMX, CTC, or ENR, and control cultures were grown without antibiotics. For each treatment, cultures were grown in triplicate to mid-log phase (A_600 _of 0.8). RNAprotect (Qiagen) was added to culture samples, and bacteria were harvested by centrifugation (10,000 × g, 10 min, 4°C). Pellets were stored at -80°C.

Total RNA from three biological replicate cultures (for untreated control culture as well as antibiotic treated cultures) was isolated using Qiagen RNeasy kit using the manufacturer's protocol. The quality and concentration of RNA was determined by Agilent Bioanalyzer.

### Microarrays and data analysis

A custom oligonucleotide microarray consisting of all the 2015 annotated open reading frames (ORFs) of *Pasteurella multocida *strain Pm70 was constructed by NimbleGen Systems. The array design included 23 perfect match (PM) and mismatch (MM) probes per target gene, and each probe was spotted twice. Reverse transcription, cDNA labeling, and hybridization were conducted by NimbleGen Sytems according to their established protocols. Briefly, three independent RNA samples (10 μg each) from untreated and antibiotic treated Pm70 were reverse transcribed using Random Hexamer (Gibco) and SuperScript II Reverse Transcriptase (Invitrogen). Biotin end-labeling of cDNA utilized Terminal Deoxynucleotidyl Transferase (Promega) with Biotin-N6-ddATP (Perkin Elmer); hybridization and washes were conducted using NimbleGen Systems protocol. Detection of hybridized biotin-labeled probe used Fluorolink Cy3 Labeled Streptavidin (Amersham Pharmacia).

Raw intensity data (single channel) were log transformed, and normalization was done by quantile normalization [28] and robust multichip analysis (RMA) algorithm [29]. The experimental design and all microarray data have been deposited in the NCBI Gene Expression Omnibus (GEO, http://www.ncbi.nlm.nih.gov/geo/, accession number GSE12779). ANOVA followed by step-down Bonferroni correction for multiple testing was conducted to identify genes with significant changes in expression relative to untreated control (p ≤ 0.05) in any group. Significant changes between control vs specific antibiotic treatments were identified by performing Dunnett's test [30]. The differences in gene expression in response to antibiotics were calculated as the ratio of treatment vs control intensities.

### Real time RT-PCR

The majority of significant changes in gene expression identified in this study were less than one-fold. Therefore, to validate changes in gene expression, we performed duplex real time quantitative RT-PCR (qPCR) of six genes: *gyrb, impA, rpsg, hpkR, recQ*, and *murA *using *gapdh *as the internal standard. The RNA template used for qPCR was the same RNA that was used for microarray hybridizations. The probes and primers for duplex RT-PCR were designed using Beacon Designer software (Additional file 4, supplemental table 3). Reactions were performed using the Platinum^® ^SYBR^® ^Green One-Step qRT-PCR Kit (Invitrogen Corporation, Carlsbad, CA). Amplification and detection of specific products were done using the iCycler iQ Real-Time PCR Detection System (Bio-Rad Laboratories, Inc., Hercules, CA). Regression analysis of the C_t _values of the test RT-PCRs was used to generate standard curves. The mean C_t _value for the GAPDH mRNA-specific reactions was used to normalize the test values. Regression analysis of the gene expression trends determined by qPCR and microarray analysis was performed in Microsoft Excel.

### Functional analysis of differentially expressed genes

Initial functional analysis of genes with significant changes in expression was done using clusters of orthologous groups (COG) classification [31]. For each COG category in the genome, the percent increase or decrease in expression of genes belonging to that category for each antibiotic treatment was calculated. The COG functional categories described for *P. multocida *genes [6] along with the description for proteins encoded by the genes at Uniprot [32] were used to identify overall themes in antibiotic response. Specific functions of *P. multocida *proteins were deduced from information available for *E. coli *orthologs at the EcoCyc database [33]. Network analysis of DE genes was done in Pathway Studio (Ariadne Genomics) as described earlier [11]. Briefly, we built interaction networks in Pathway Studio with proteins of interest including the upstream regulators and downstream targets. In the interaction networks, different colors were used for nodes to indicate significant increase (red), decrease (green), or no change (pink) in gene expression in response to sub-MIC of antibiotic. Entities in the interaction map that were not present in the Pm70 genome were shown in gray color.

## List of abbreviations used

MIC: minimum inhibitory concentration; CTC: chlortetracycline; AMX: amoxicillin; ENR: enrofloxacin; BHI: brain heart infusion; COG: Clusters of Orthologous Groups; DE: differentially expressed; recA: recombinase A; gyrB: DNA gyrase B.

## Competing interests

The authors declare that they have no competing interests.

## Authors' contributions

BN cultured Pm70, did RNA isolations, data analysis and network modeling and wrote the draft of the manuscript. LAS carried out the qPCR validations and contributed to the methods and results of this aspect in the manuscript. ML and SCB conceived and coordinated this study and helped draft the manuscript. All authors read and approved the final manuscript.

## Supplementary Material

Additional file 1 Significant changes identified in *P. multocida *gene expression with AMX, CTC and ENR. Click here for file

Additional file 2 Supplemental table 2. Significant changes identified in *P. multocida *gene expression common to more than one antibiotic. Click here for file

Additional file 3 **Supplemental figures. Interaction networks**. The network diagrams show changes in gene expression in response to sub-MIC. Red and green indicate significant increase and decrease in expression respectively. Genes whose expression did not change (pink) and genes in the network from other bacteria (gray) are also included. *Supplemental figure 1*. fnr regulated genes with CTC administration. *Supplemental figure 2*. Interaction network with genes involved in SOS response with AMX administration. *Supplemental figure 3*. Interaction network with genes involved in SOS response with CTC administration. Click here for file

Additional file 4 **Supplemental table 3. Quantitative RT-PCR probes and primers**. The sequences of primers used for qPCR validations of selected gene expression changes in microarray. Click here for file
