# Supplementary material for: The transcriptional response of Pasteurella multocida to three classes of antibiotics
Source: BMC Genomics. 2009 Jul 14;10(Suppl 2):S4. doi: 10.1186/1471-2164-10-S2-S4 (PMC2966327; doi:10.1186/1471-2164-10-S2-S4)
Supplement: Additional file 2 — Supplemental table 2. Significant changes identified in P. multocida gene expression common to more than one antibiotic. [file 1471-2164-10-S2-S4-S2.doc]

**Supplemental t**able 2. Some significant changes in gene expression common to more than one antibiotic

| GeneID | Product Name | Locus | AMX* | CTC* | ENR* |
| --- | --- | --- | --- | --- | --- |
|  | **TCA cycle enzymes and ATP synthesis** |  |  |  |  |
| 1243625 | Hypothetical protein PM0278 | sucB | 1.15 | 1.22 | -0.77 |
| 1244240 | Dihydrolipoamide dehydrogenase | lpdA | -0.93 | -0.90 | -0.78 |
| 1244242 | Pyruvate dehydrogenase subunit E1 | aceE | -0.91 | -0.87 | 1.12 |
| 1244678 | Na(+)-translocating NADH-quinone reductase subunit D | - | ns | -0.73 | 1.26 |
| 1244679 | Na(+)-translocating NADH-quinone reductase subunit E | nqrE | -0.88 | -0.68 | 1.15 |
| 1244834 | F0F1 ATP synthase subunit I | - | -0.84 | -0.81 | 1.78 |
| 1244835 | F0F1 ATP synthase subunit A | atpB | ns | -0.81 | 1.73 |
| 1244836 | F0F1 ATP synthase subunit C | atpE | -0.91 | -0.87 | 1.55 |
| 1244837 | F0F1 ATP synthase subunit B | atpF | 1.06 | -0.89 | 1.54 |
| 1244838 | F0F1 ATP synthase subunit delta | atpH | ns | -0.86 | 1.62 |
| 1244839 | F0F1 ATP synthase subunit alpha | atpA | ns | -0.89 | 1.43 |
| 1244840 | F0F1 ATP synthase subunit gamma | atpG | -0.89 | -0.91 | 1.62 |
| 1244842 | F0F1 ATP synthase subunit epsilon | atpC | -0.87 | -0.87 | ns |
|  | **Transporters** |  |  |  |  |
| 1243475 | Iron-dicitrate transporter ATP-binding subunit | fecE | -0.69 | ns | -0.59 |
| 1243477 | Iron-dicitrate transporter permease subunit | fecC | -0.79 | ns | -0.55 |
| 1244089 | Molybdate transporter ATP-binding protein | modC | -0.55 | -0.56 | -0.59 |
| 1244090 | Molybdate ABC transporter permease protein | modB | -0.77 | -0.69 | ns |
| 1244237 | Putative molybdenum transport ATP-binding protein ModF | modF | -0.62 | -0.63 | ns |
| 1243607 | PotD | potD_1 | ns | ns | 3.28 |
| 1244154 | Putrescine transporter | potE | -0.56 | -0.66 | -0.55 |
| 1244731 | PotD | potD_3 | -0.79 | ns | 1.57 |
| 1244674 | RbsC | rbsC_3 | -0.56 | -0.74 | -0.40 |
| 1243501 | Ribose ABC transporter permease protein | rbsC | -0.80 | -0.83 | -0.41 |
| 1243502 | D-ribose transporter ATP binding protein | rbsA_1 | -0.75 | -0.55 | -0.35 |
|  | **Nucleotide biosynthesis** |  |  |  |  |
| 1243368 | Phosphoribosylaminoimidazole synthetase | purM | 1.22 | 1.35 | 1.29 |
| 1243569 | Bifunctional phosphoribosylaminoimidazolecarboxamide formyltransferase | purH | -0.65 | -0.76 | ns |
| 1243966 | Phosphoribosylaminoimidazole carboxylase catalytic subunit | purE | -0.64 | ns | -0.69 |
| 1243967 | Phosphoribosylaminoimidazole carboxylase ATPase subunit | purK | -0.68 | -0.65 | -0.66 |
| 1244048 | Amidophosphoribosyltransferase | purF | -0.79 | 1.06 | -0.84 |
| 1244064 | Ribonucleotide-diphosphate reductase subunit alpha | nrdA | -0.94 | -0.86 | 1.61 |
| 1244066 | Ribonucleotide-diphosphate reductase subunit beta | nrdB | ns | ns | 1.69 |
| 1244149 | Cytidylate kinase | cmk | -0.85 | -0.89 | 1.26 |
| 1244285 | Adenylosuccinate synthetase | purA | -0.90 | ns | 1.36 |
| 1245020 | Thymidylate kinase | tdk | -0.83 | -0.96 | 1.27 |
| 1245219 | CTP synthetase | pyrG | -0.74 | -0.83 | 1.44 |
| 1245333 | Uridylate kinase | pyrH | 1.17 | -0.90 | 1.82 |
|  | **Fatty acid biosynthesis** |  |  |  |  |
| 1244720 | Hypothetical protein PM1373 | fbaA | ns | ns | 2.05 |
| 1243686 | 3-oxoacyl-(acyl carrier protein) synthase I | fabB | -0.88 | -0.87 | ns |
| 1243983 | Acetyl-CoA carboxylase subunit beta | accD | -0.85 | ns | 1.32 |
| 1244039 | Acetyl-CoA synthetase | acsA | -0.50 | -0.49 | -0.67 |
| 1244054 | Long-chain-fatty-acid--CoA ligase | fadD_1 | ns | ns | 1.75 |
| 1244439 | Acetyl-CoA carboxylase biotin carboxyl carrier protein subunit | accB | -0.91 | ns | 1.49 |
| 1245262 | Acyl carrier protein S-malonyltransferase | fabD | 1.08 | ns | 1.33 |
| 1245263 | 3-ketoacyl-(acyl-carrier-protein) reductase | fabG | 1.09 | ns | 1.29 |
| 1245342 | FabZ | fabZ | ns | -0.87 | 1.38 |
|  | **Ribosomal proteins** |  |  |  |  |
| 1244148 | 30S ribosomal protein S1 | rpsA | -0.88 | -0.72 | ns |
| 1244524 | 50S ribosomal protein L9 | rplI | ns | -0.82 | 1.74 |
| 1244525 | 30S ribosomal protein S18 | rpsR | ns | -0.74 | 1.62 |
| 1244645 | 50S ribosomal protein L19 | rplS | -0.90 | -0.74 | 1.08 |
| 1244701 | 30S ribosomal protein S12 | rpsL | ns | ns | 1.55 |
| 1244702 | 30S ribosomal protein S7 | rpS7 | 1.11 | -0.93 | 1.69 |
| 1244736 | 50S ribosomal protein L17 | rplQ | -0.92 | -0.62 | 1.08 |
| 1244738 | 30S ribosomal protein S4 | rpsD | ns | -0.82 | 1.29 |
| 1244739 | 30S ribosomal protein S11 | rpS11 | -0.95 | -0.78 | 1.10 |
| 1244740 | 30S ribosomal protein S13 | rpsM | -0.89 | -0.77 | 1.31 |
| 1244741 | 50S ribosomal protein L36 | rpmJ | ns | -0.82 | 1.44 |
| 1244743 | 50S ribosomal protein L15 | rplO | -0.90 | -0.76 | 1.15 |
| 1244744 | 50S ribosomal protein L30 | rpmD | ns | -0.83 | 1.32 |
| 1244745 | 30S ribosomal protein S5 | rpsE | -0.80 | -0.62 | 1.34 |
| 1244746 | 50S ribosomal protein L18 | rplR | ns | -0.85 | 1.19 |
| 1244747 | 50S ribosomal protein L6 | rplF | -0.89 | -0.62 | 1.20 |
| 1244748 | 30S ribosomal protein S8 | rpsH | 1.13 | -0.74 | 1.51 |
| 1244749 | 30S ribosomal protein S14 | rpsN | -0.88 | -0.77 | 1.11 |
| 1244750 | 50S ribosomal protein L5 | rplE | -0.95 | -0.72 | 1.20 |
| 1244751 | 50S ribosomal protein L24 | rplX | ns | -0.86 | 1.50 |
| 1244752 | 50S ribosomal protein L14 | rplN | ns | -0.82 | 1.14 |
| 1244754 | 50S ribosomal protein L29 | rpmC | -0.86 | -0.86 | ns |
| 1244755 | 50S ribosomal protein L16 | rplP | -0.81 | -0.85 | 1.07 |
| 1244756 | 30S ribosomal protein S3 | rpsC | -0.95 | -0.80 | ns |
| 1244757 | 50S ribosomal protein L22 | rplV | -0.83 | -0.75 | -0.88 |
| 1244758 | 30S ribosomal protein S19 | rpsS | -0.86 | -0.73 | ns |
| 1244759 | 50S ribosomal protein L2 | rplB | -0.86 | -0.79 | ns |
| 1244760 | 50S ribosomal protein L23 | rplW | -0.92 | -0.73 | 1.16 |
| 1244762 | 50S ribosomal protein L3 | rplC | -0.95 | -0.82 | ns |
| 1244763 | 30S ribosomal protein S10 | rpsJ | -0.83 | -0.70 | ns |
| 1245085 | 50S ribosomal protein L7/L12 | rplL | -0.89 | -0.81 | 1.10 |
| 1245086 | 50S ribosomal protein L10 | rplJ | -0.87 | -0.84 | 1.09 |
| 1245331 | 30S ribosomal protein S2 | rpsB | -0.91 | -0.87 | 1.28 |
| 1245362 | 30S ribosomal protein S16 | rpsP | -0.87 | -0.77 | 1.06 |
| 1245358 | histidyl-tRNA synthetase | hisS | ns | -0.81 | 1.61 |

* Ratio of treatment/control normalized intensities

ns: no significant change in gene expression with sub-MIC of antibiotic
