# Supplementary figures and images for: The transcriptional response of Pasteurella multocida to three classes of antibiotics
Source: BMC Genomics. 2009 Jul 14;10(Suppl 2):S4. doi: 10.1186/1471-2164-10-S2-S4 (PMC2966327; doi:10.1186/1471-2164-10-S2-S4)

## Slide 1
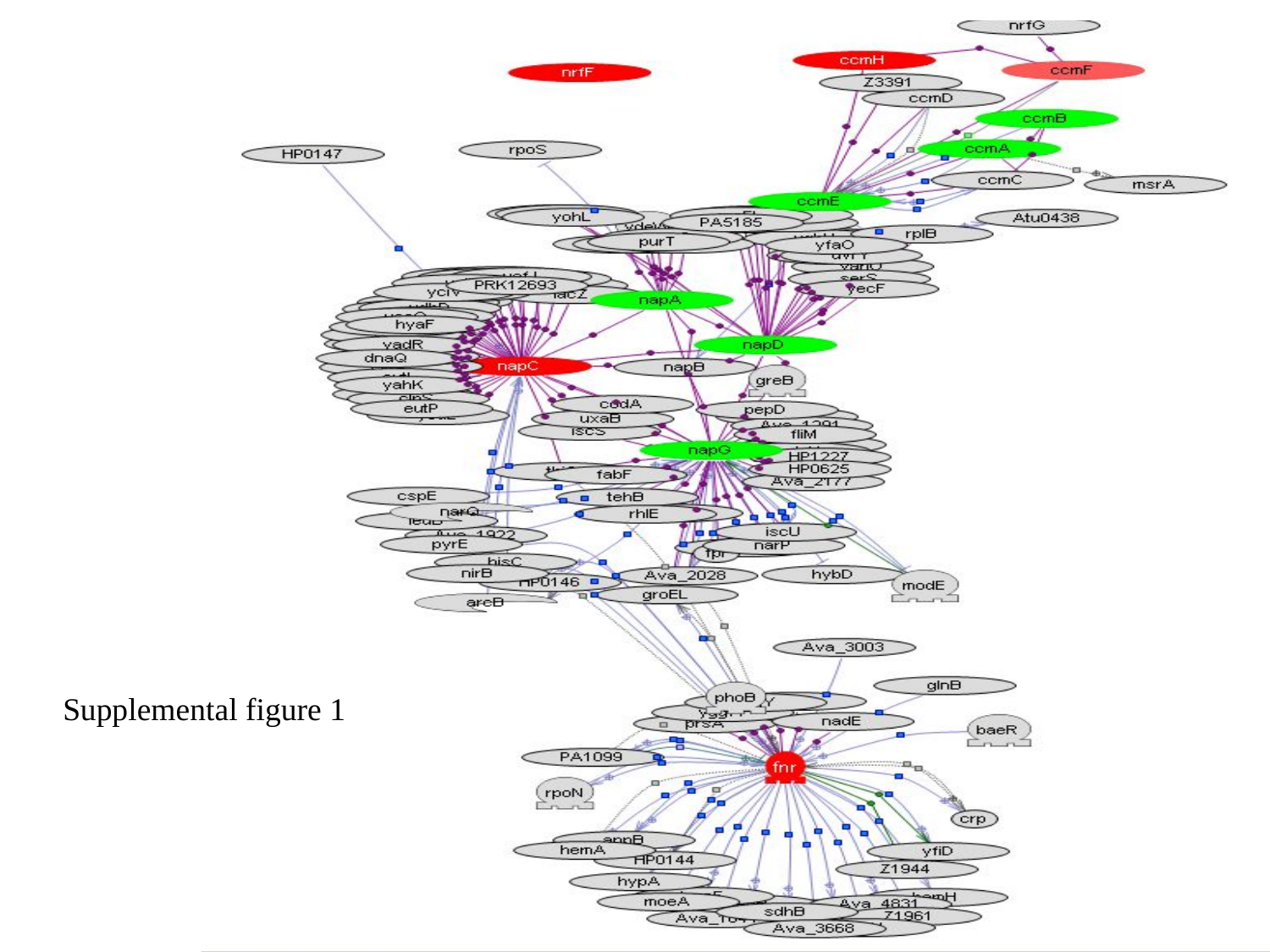

Supplemental figure 1

## Slide 2
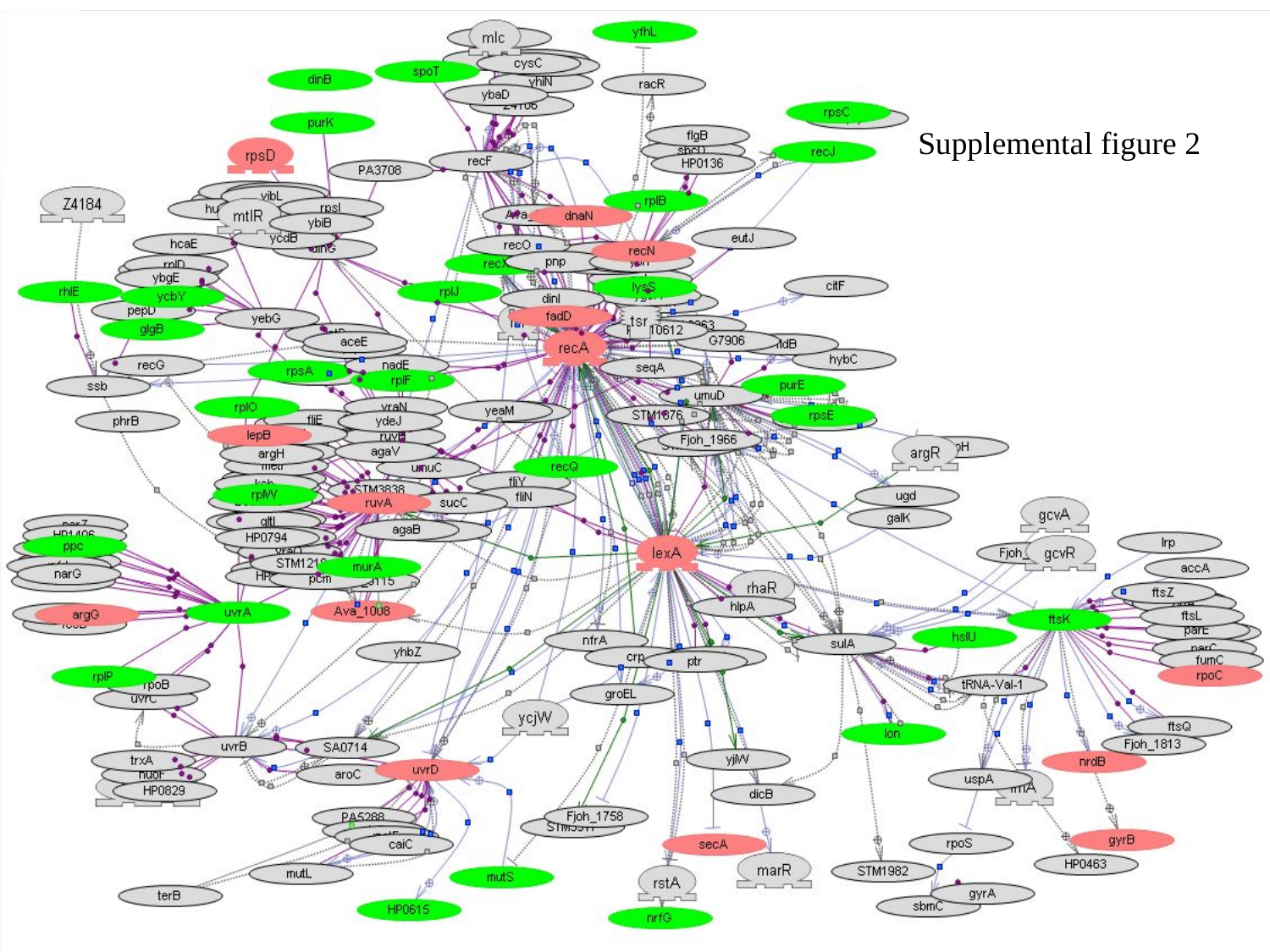

Supplemental figure 2

## Slide 3
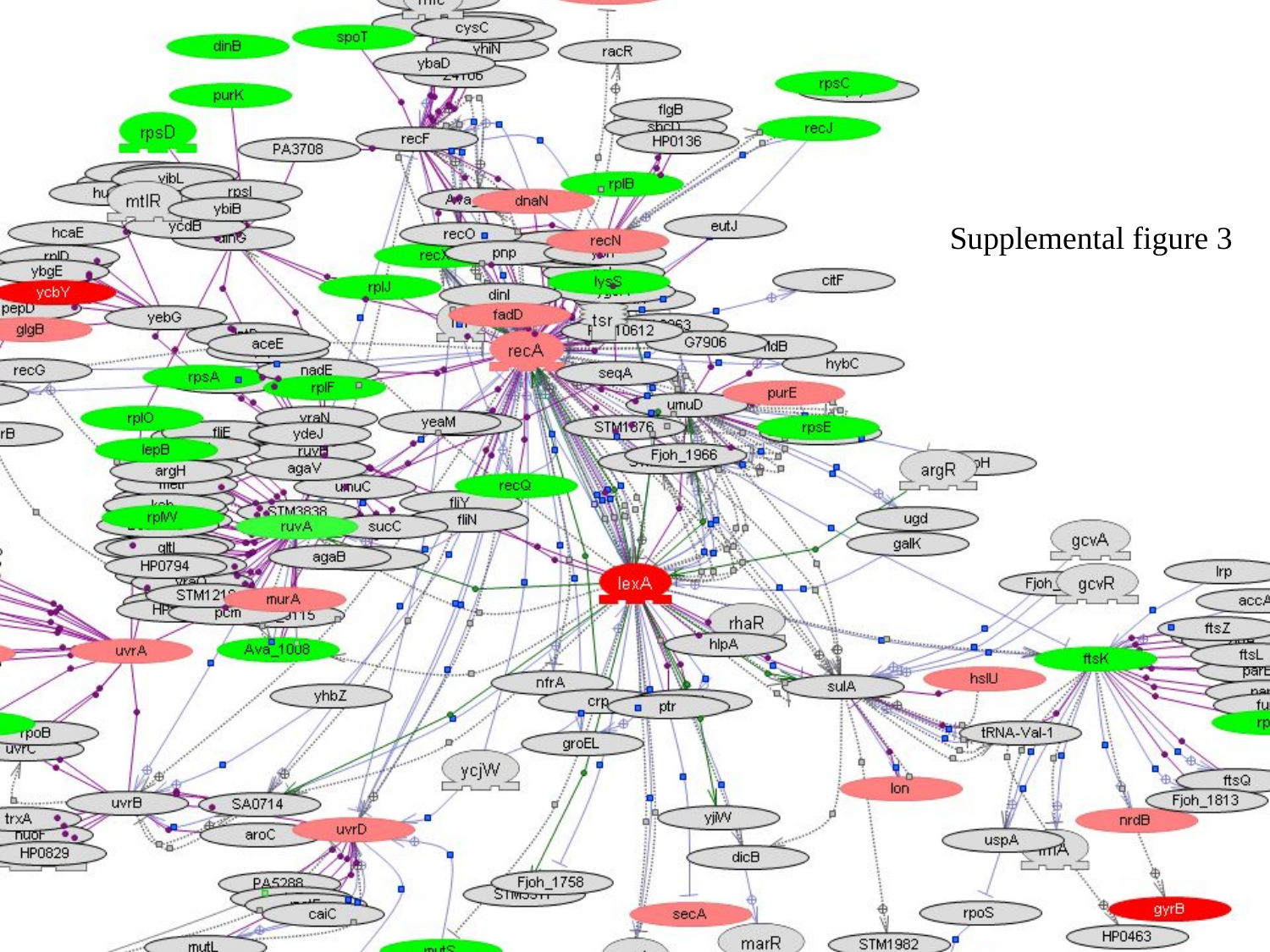

Supplemental figure 3

Supplement: Additional file 3 — Supplemental figures. Interaction networks. The network diagrams show changes in gene expression in response to sub-MIC. Red and green indicate significant increase and decrease in expression respectively. Genes whose expression did not change (pink) and genes in the network from other bacteria (gray) are also included. Supplemental figure 1. fnr regulated genes with CTC administration. Supplemental figure 2. Interaction network with genes involved in SOS response with AMX administration. Supplemental figure 3. Interaction network with genes involved in SOS response with CTC administration. [file 1471-2164-10-S2-S4-S3.ppt]
