# Supplementary material for: The transcriptional response of Pasteurella multocida to three classes of antibiotics
Source: BMC Genomics. 2009 Jul 14;10(Suppl 2):S4. doi: 10.1186/1471-2164-10-S2-S4 (PMC2966327; doi:10.1186/1471-2164-10-S2-S4)
Supplement: Additional file 4 — Supplemental table 3. Quantitative RT-PCR probes and primers. The sequences of primers used for qPCR validations of selected gene expression changes in microarray. [file 1471-2164-10-S2-S4-S4.doc]

Supplemental table 3. Quantitative RT-PCR probes and primers

RNA target Probe/primer Sequence

GAPDH Probe 5′-(HEX)-AACTATCCGTGTCACTGCTGAGCGTG-(TAMRA)-3′

F 5′-AGTAAAAGATGGCAACTTAGTGGTAA-3′

R 5′-CAGTCGCTTCAACGGCAATT-3′

GyrB Probe 5′-(FAM)-TTCATTCATCACCGATTCCACCGCACTT-(TAMRA)-3′

F 5′-ATCCTAAATTCTCCTCGCAAACG-3′

R 5′-TTTGGCATCGGACGGGTTT-3′

hpkR Probe 5′-(FAM)-CACCGCAACGCCGACATTACACCGAT-(TAMRA)-3′

F 5′-TGAGGTAAACAATGCCGATTTAGC-3′

R 5′-ATCCCTTGATTCACTAAGTCCATCAT-3′

ImpA Probe 5′-(FAM)-CGCCCACAACAAACCTGCTTTATTCGTG-(TAMRA)-3′

F 5′-AAAGCTGGATCTCAATCTTTACTGTG-3′

R 5′-TCAATTCCCCAAGCAAGCATATTC-3′

MurZ Probe 5′-(FAM)-ACTATGCTCACAACCGCCTAAACGCTCA-(TAMRA)-3′

F 5′-GCCAAAGGGAAAACCATCATTG-3′

R 5′-TTTACATTCAATGCGTCCACCTG-3′

recQ Probe 5′-(FAM)-ACCGCTTCCGCAGGCAAATCATCACG-(TAMRA)-3′

F 5′-CTTACTATCAAGAAACAGGACGAGC-3′

R 5′-TTTATGTAGCCAAGCGTAATCTGC-3′

RpS7 Probe 5′-(FAM)-AGTAGAACCACCCACACGGCGAGA-(TAMRA)-3′

F 5′-CACATTAGCACAACGTACAGGTAA-3′

R 5′-GTACTGGACGAACTTCTACAGGTA-3′
